# Long non-coding RNA DANCR regulates proliferation and apoptosis of chondrocytes in osteoarthritis via miR-216a-5p-JAK2-STAT3 axis

**DOI:** 10.1042/BSR20181228

**Published:** 2018-12-14

**Authors:** Lei Zhang, Pin Zhang, Xiangyi Sun, Liwu Zhou, Jianning Zhao

**Affiliations:** 1 Department of Orthopedics, Jinling Hospital, Nanjing University, School of Medicine, Nanjing 210002, China; 2 Department of Orthopedics, Jinling Clinical Medical College of Nanjing Medical University, Nanjing 210002, China

**Keywords:** DANCR, JAK2, miR-216a-5p, Osteoarthritis, STAT3

## Abstract

Osteoarthritis (OA) is one of the most common chronic joint disease. Long non-coding RNAs (lncRNAs) have been confirmed to play important roles in a variety of diseases including OA. However, the underlying mechanism of lncRNA differentiation antagonizing non-protein coding RNA (DANCR) in OA has not been well elucidated. The expression of DANCR in cartilage tissues from OA patients was detected using quantitative real-time PCR. After cell transfection, the effects of DANCR inhibition on the proliferation, apoptosis and inflammatory factors of OA chondrocytes were detected using Cell Counting Kit-8 assay and flow cytometry assay. Novel target of DANCR was then identified through bioinformatics analysis and confirmed by luciferase reporter assay and RNA immunoprecipitation assay. The expression of DANCR was significantly increased in OA patients. Function assays demonstrated that DANCR suppression inhibited the proliferation, inflammation, and promoted apoptosis of chondrocytes cells. Additionally, DANCR regulated survival of OA chondrocytes through acting as a competitive endogenous RNA for miR-216a-5p. Furthermore, JAK2 was a direct target of miR-216a-5p, and DANCR regulated the JAK2/STAT3 signal pathway through miR-216a-5p in OA chondrocytes. In the present study, we concluded that DANCR promoted the proliferation, inflammation, and reduced cell apoptosis in OA chondrocytes through regulating miR-216a-5p/JAK2/STAT3 signaling pathway, indicating DANCR might be a useful biomarker and potential therapeutic target for OA treatment.

## Introduction

Osteoarthritis (OA) is a common chronic joint disease, which is mainly characterized by proteolytic degradation of the cartilage and synovial inflammation [1]. The incidence rate of OA increases with age, so it has become the main cause of pain, disability, and shortening of adult working life around the world [2]. Current therapeutic options for OA are aimed at pain reduction and symptom control. However, there is no approved treatment that can modify the disease progression [3]. To improve the therapeutic options available for patients with OA, it is necessary to explore the pathophysiology and regulatory mechanism of OA.

Long non-coding RNAs (lncRNAs) are a class of transcripts (longer than 200 nucleotides in length) without the function of encoding proteins [4]. LncRNAs have been shown to play essential roles in multiple biological processes [5–7]. In recent years, increasing evidences have demonstrated that dysregulation of lncRNA is closely associated with diverse diseases including OA [8–10]. For example, lncRNA-CIR was significantly up-regulated in OA patients and overexpression of lncRNA-CIR promoted the degradation of the extracellular matrix of chondrocyte in OA [11].

Differentiation antagonizing non-protein coding RNA (DANCR) is located in human chromosome 4q12, and is reported to play an important role in a variety of cellular biological processes. For example, Yuan et al. suggested that lncRNA DANCR increased stemness features of hepatocellular carcinoma by derepression of catenin β-1 (CTNNβ-1) [12]. Zhang et al. revealed that lncRNA DANCR promoted the proliferation and chondrogenic differentiation through up-regulating Smad3 and STAT3 in human mesenchymal stem cells [13]. However, the functional role and underlying molecular mechanisms of DANCR involved in OA progression remain uncertain.

In the present study, we examined the expression of DANCR in cartilage samples from OA patients and healthy subjects, and then investigated the functional role of DANCR in OA chondrocytes.

## Materials and methods

### Clinical samples

30 OA cartilage tissues were collected from patients who underwent total knee replacement surgery. 20 normal cartilage tissues were obtained from patients with femoral neck fracture who underwent total hip replacement surgery without OA or rheumatoid arthritis history. All patients signed written informed consent in the present study. The present study was approved by the Research Ethics Committee of Jinling Hospital.

### Cell culture

The chondrocytes were isolated from OA cartilage tissues as previously described [14]. Chondrocytes were cultured in the Dulbecco’s modified Eagle’s medium (DMEM) containing 10% FBS in a humidified atmosphere at 37˚C with 5% CO_2_.

### RNA extraction and quantitative real-time PCR

Total RNAs were isolated from human cartilage tissues and cultured chondrocytes using the Trizol reagent (Invitrogen, Carlsbad, U.S.A.), and then reversed transcribed. Quantitative real-time PCR (qRT-PCR) was carried out using the SYBR Green PCR Kit (Takara, Japan) on the ABI 7500 Fast Real-Time PCR system (Applied Biosystems, CA, U.S.A.) following the manufacturer’s instructions. Relative miRNA expression was normalized to the U6 expression, and the expression of mRNA and lncRNA was normalized to GAPDH. The relative gene expression was calculated using the 2^−ΔΔ*C*^_t_ method. The primer sequences were showing as follows: DANCR: 5′-GCGCCACTATGTAGCGGGTT-3′ (forward) and 5′-TCAATGGCTTGTGCCTGTAGTT-3′ (reverse), MiR-216a-5p: 5′-GGGCTGGACCGAGAGAGTTT-3′ (forward) and 5′-CCTTGTACGTGGTGGGATTGA-3′ (reverse), JAK2: 5′-GGGAGGTGGTCGCTGTAAAA-3′ (forward) and 5′-ACCAGCACTGTAGCACACTC-3′ (reverse), Interleukin-6 (IL-6): 5′-GACTGATGTTGCTGACAGCCACTGC-3′ (forward) and 5′-TAGCCACTGCTTCTGTGACTCTAACT-3′ (reverse), IL-8: 5′-AAACCACCGGAAGGAACCAT-3′ (forward) and 5′-GCCAGCTTGGAAGTCATGT-3′ (reverse).

### Cell transfection

The specific interference sequence for DANCR (si-DANCR) was constructed at Genechem (Shanghai, China), and then transfected into chondrocytes. The sequences were showing as follows: si-DANCR-1: 5′-UCGGAGGUGGAUUCUGUUA-3′, si-DANCR-2: 5′-AGCCAACTATCCCTTCAGT-3′. MiR-216a-5p mimics, miR-216a-5p inhibitors, and respective negative control miRNA were synthesized by GenePharma (Shanghai, China). Transfection was performed using Lipofectamine 3000 (Invitrogen, U.S.A.) according to the manufacturer’s instructions.

### Cell counting kit-8 assay

Cell proliferation was assessed by Cell Counting Kit-8 (CCK-8) assay. The cells were seeded in a 96-well plate at a density of 2 × 103 cells/well. After incubation for 24, 48, 72, and 96 h, CCK-8 solution (DOJINDO, Tokyo, Japan) was added to each well. The absorbance was measured at the wavelength of 450 nm using a microplate reader.

### Cell apoptosis assay

Cell apoptosis was determined using the Annexin V-FITC/PI apoptosis detection kit (BD Biosciences, U.S.A.). The cells were harvested after transfection for 48 h, washed twice with PBS, and then stained with Annexin V-FITC and PI for 15 min at room temperature in the dark. Apoptotic cells were analyzed using FACS flow cytometry (BD Biosciences, U.S.A.).

### Luciferase reporter assay

The amplified DNA sequences were cloned to the pmirGLO dual-luciferase vector (Promega, Madison, WI, U.S.A.) to form the wild-type DANCR 3′UTR (DANCR-WT) and mutated DANCR 3′-UTR (DANCR-MUT) reporter vectors. Human chondrocytes were plated in 24-well plates overnight and then transfected with the wild-type or mutated reporter vectors and miR-216a-5p mimic using Lipofectamine 3000 (Invitrogen, Carlsbad, CA, U.S.A.). Similarly, the JAK2-WT and JAK2-MUT reporter vectors were synthesized and transfected into chondrocytes. 48 h after transfection, the luciferase activity was measured by the dual-luciferase reporter assay system (Promega, Madison, WI, U.S.A.).

### Western blot assay

Chondrocytes were lysed on ice with RIPA protein extraction reagent and then protein concentrations were measured using the BCA Protein Assay Kit (Beyotime, Shanghai, China). Protein fractions were separated by 10% SDS/PAGE and then transferred onto PVDF membrane (Millipore, Bedford, MA, U.S.A.). After blocked with 5% non-fat milk for 1 h at room temperature, membranes were incubated with specific primary antibodies (Abcam, Cambridge, U.K.) at 4°C overnight. The membranes were then incubated with HRP-conjugated secondary antibodies at room temperature for 1 h. All bands were finally analyzed using the ECL detection system (Pierce Biotechnology, Rockford, U.S.A.) according to manufacturer’s instructions.

### RNA immunoprecipitation assay

RNA immunoprecipitation (RIP) assay was performed by the EZ-Magna RIP RNA-Binding Protein Immunoprecipitation Kit (Millipore, Bedford, MA, U.S.A.) following the manufacturer’s protocol. Chondrocytes were collected and resuspended in RIP lysis buffer. Then, cell lysate was incubated with RIP buffer containing magnetic beads conjugated with anti-Argonaute2 (anti-Ago2) or anti-IgG (negative control) overnight at 4°C. RNA was purified from RNA–protein complexes bound to the beads. The relative enrichment of DANCR and miR-216a-5p were measured by qRT-PCR analysis.

### Statistical analysis

All the data were presented as the mean ± S.D. from three independent experiments as indicated. Statistical analysis was carried out using SPSS 20.0 (SPSS, Chicago, IL, U.S.A.). Differences between groups were analyzed by two-tailed Student’s *t*-test, χ^2^ test or one-way ANOVA analysis. *P*<0.05 was considered statistically significant.

## Results

### DANCR expression was significantly increased in OA patients

**Figure 1:**
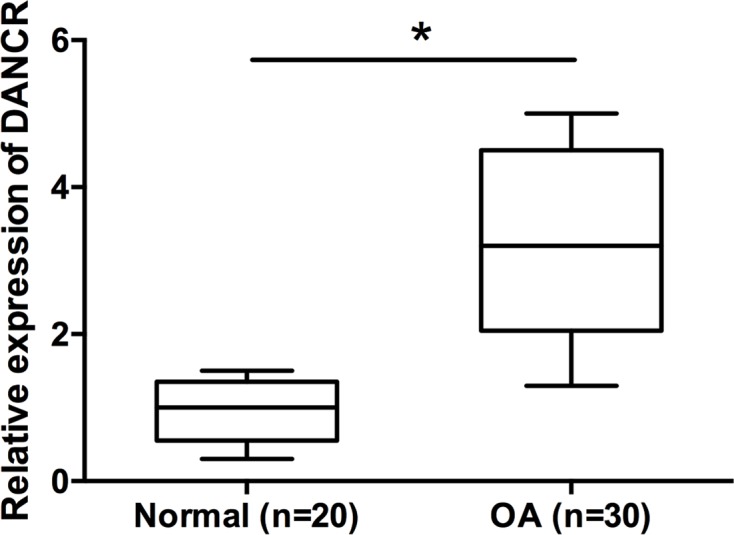
LncRNA DANCR expression was significantly increased in OA patients The expression of DANCR in articular cartilage samples from normal patients and OA patients was detected by qRT-PCR. Data are shown as mean ± S.D. ^*^P<0.05.

To explore whether DANCR was altered in OA, the expression level of DANCR was detected using qRT-PCR assay in 30 OA patients and 20 normal patients. As shown in Figure 1, the DANCR expression was significantly up-regulated in the OA group compared with the normal group (*P*<0.05).

### DANCR suppression inhibited cell proliferation, inflammation, and promoted cell apoptosis in OA chondrocytes

**Figure 2:**
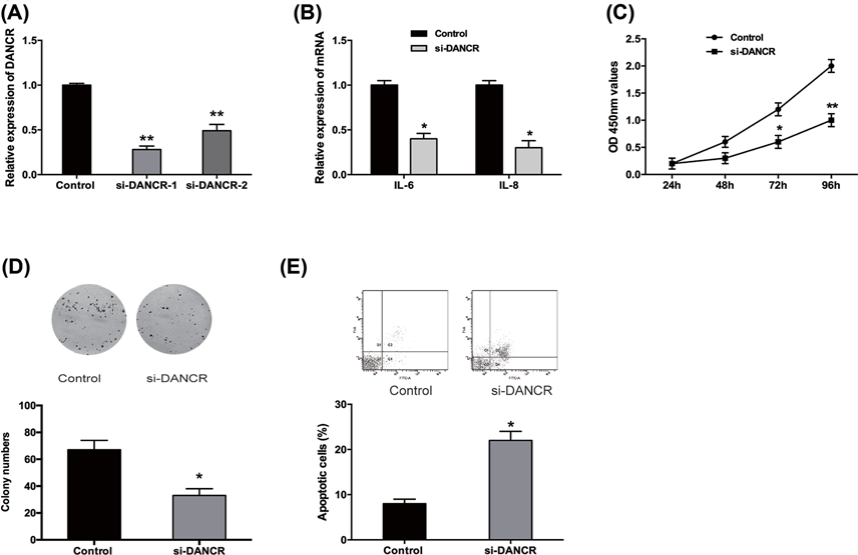
DANCR inhibition suppressed the proliferation, inflammation, and promoted apoptosis in OA chondrocytes (**A**) The relative expression of DANCR in OA chondrocytes with DANCR inhibition. (**B**) The effect of DANCR inhibition on expression of inflammatory factors (IL-6 and IL-8) in OA chondrocytes was detected by qRT-PCR. (**C** and **D**) The effect of DANCR inhibition on proliferation and colony formation of OA chondrocytes was detected by CCK-8 assay colony formation assay. (**E**) The effect of DANCR inhibition on apoptosis of OA chondrocytes was detected by flow cytometric assay. Data are shown as mean ± S.D. ^*^P<0.05, ^**^P<0.01.

To investigate the effect of DANCR on OA chondrocytes, DANCR was decreased in chondrocytes through cell transfection (Figure 2A, *P*<0.05). qRT-PCR was used to determine the expression levels of inflammatory factors, including IL-6 and IL-8. Results showed that IL-6 and IL-8 were significantly inhibited in chondrocytes by down-regulation of DANCR (Figure 2B, *P*<0.05). By performing CCK-8 assay and colony assay, we found that down-regulation of DANCR suppressed the proliferation viability of chondrocytes cells (Figure 2C,D, *P*<0.05). In addition, we analyzed the difference in cell apoptosis after DANCR inhibition by flow cytometric assay. The results showed that down-regulation of DANCR induced cell apoptosis in chondrocytes (Figure 2E, *P*<0.05). These results indicated that DANCR could promote proliferation, inflammatory cytokine expression, and reduce apoptosis of chondrocytes.

### DANCR directly interacted with miR-216a-5p in OA chondrocytes

**Figure 3:**
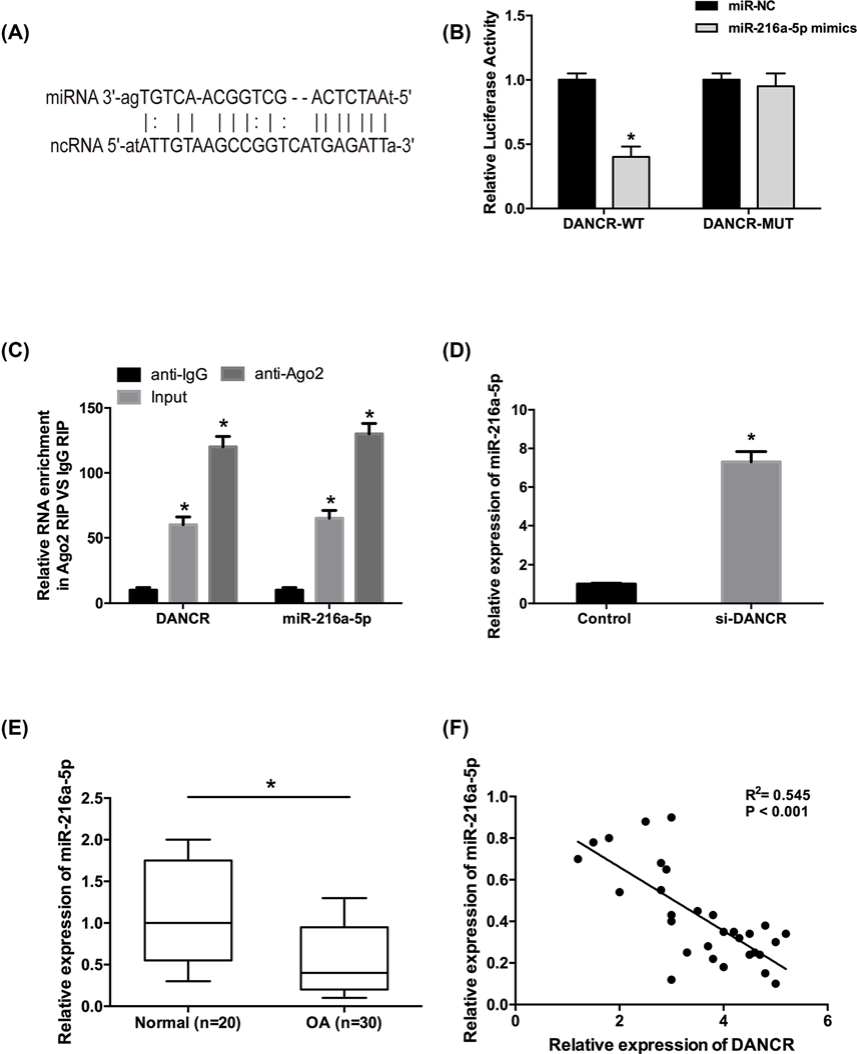
DANCR directly interacted with miR-216a-5p in OA chondrocytes (**A**) A putative miR-216a-5p target site in the 3′-UTR of DANCR mRNA. (**B**) Luciferase reporter assay demonstrated that miR-216a-5p mimics significantly decreased the luciferase activity of DANCR-WT in OA chondrocytes. (**C**) The interaction between DANCR and miR-216a-5p was detected by RIP assay. (**D**) DANCR inhibition increased the relative expression of miR-216a-5p in OA chondrocytes. (**E**) The relative expression of miR-216a-5p was significantly down-regulated in OA patients compared with normal patients. (**F**) The inverse relationship was observed between DANCR and miR-216a-5p expression in OA cartilage tissues. Data are shown as mean ± S.D. ^*^P<0.05.

Recent studies indicated that lncRNAs could act as a competitive endogenous RNA (ceRNA) or molecular sponges for specific miRNAs, and in turn regulating their biological functions. To further investigate the underlying mechanism of DANCR in OA, the online software (starBase v2.0) was used to research for the miRNAs interacted with DANCR. Interestingly, we found that miR-216a-5p contained putative binding sites with DANCR (Figure 3A). To confirm whether miR-216a-5p was a direct target of DANCR in chondrocytes, a dual-luciferase reporter assay was performed. The results showed that miR-216a-5p mimics significantly decreased the luciferase activity of DANCR-WT reporter vector but not the DANCR-MUT reporter vector (Figure 3B, *P*<0.05). In addition, an anti-Ago2 RIP assay was also applied to identify the endogenous interaction between DANCR and miR-216a-5p in chondrocytes. The results showed that DANCR and miR-216a-5p were preferentially enriched in the Ago2 pellet compared with the negative control (Figure 3C, *P*<0.05). Furthermore, DANCR inhibition strongly increased the expression of miR-216a-5p in chondrocytes (Figure 3D, *P*<0.05).

Moreover, we measured the expression of miR-216a-5p in cartilage tissues, and we found that miR-216a-5p expression was significantly decreased in the OA group compared with the normal group (Figure 3E, *P*<0.05). An inverse correlation was found between DANCR and miR-216a-5p expressions in 30 OA patients by Pearson’s correlation analysis (Figure 3F, *P*<0.05). These results indicated that miR-216a-5p was a direct inhibitory target of DANCR in OA chondrocytes.

### DANCR regulated proliferation, inflammation, and apoptosis of OA chondrocytes through miR-216a-5p

**Figure 4:**
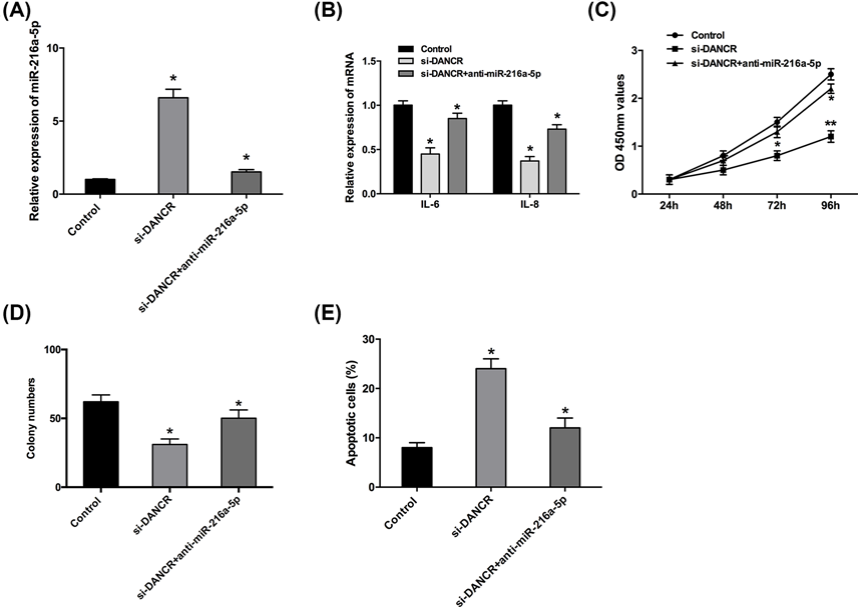
DANCR regulated inflammation, proliferation, and apoptosis of OA chondrocytes through miR-216a-5p (**A**) MiR-216a-5p inhibitors could decrease the miR-216a-5p expression in DANCR-down-regulation chondrocytes. (**B**) MiR-216a-5p inhibitors abolished the inhibition of IL-6 and IL-8 expression in OA chondrocytes induced by DANCR inhibition. (**C** and **D**) MiR-216a-5p inhibitors abolished the inhibition of proliferation and colony formation in OA chondrocytes induced by DANCR suppression. (**E**) MiR-216a-5p inhibitors abolished the promotion of apoptosis in OA chondrocytes induced by DANCR inhibition. Data are shown as mean ± S.D. ^*^P<0.05, ^**^P<0.01.

To investigate whether miR-216a-5p was involved in the regulation of DANCR in OA chondrocytes, we transfected the miR-216a-5p inhibitors into DANCR-suppression chondrocytes. As shown in Figure 4A, miR-216a-5p inhibitors could decrease miR-216a-5p expression in DANCR-inhibition chondrocytes (*P*<0.05). qRT-PCR showed that the inhibitory effects on IL-6 and IL-8 expression levels induced by DANCR inhibition in OA chondrocytes could be partly abolished by the introduction of miR-216a-5p inhibitors (Figure 4B, *P*<0.05). Furthermore, CCK-8 and colony formation assays revealed that the decrease of cell proliferation and colony formation ability induced by DANCR inhibition was reversed by the miR-216a-5p inhibitors (Figure 4C,D, *P*<0.05). Meanwhile, DANCR suppression led to the promotion of apoptosis in chondrocytes, which was reversed by the miR-216a-5p inhibitors (Figure 4E, *P*<0.05). These results revealed that DANCR promoted inflammation, proliferation, and reduced the apoptosis of OA chondrocytes through down-regulating miR-216a-5p expression.

### JAK2 was a direct target of miR-216a-5p in OA chondrocytes

**Figure 5:**
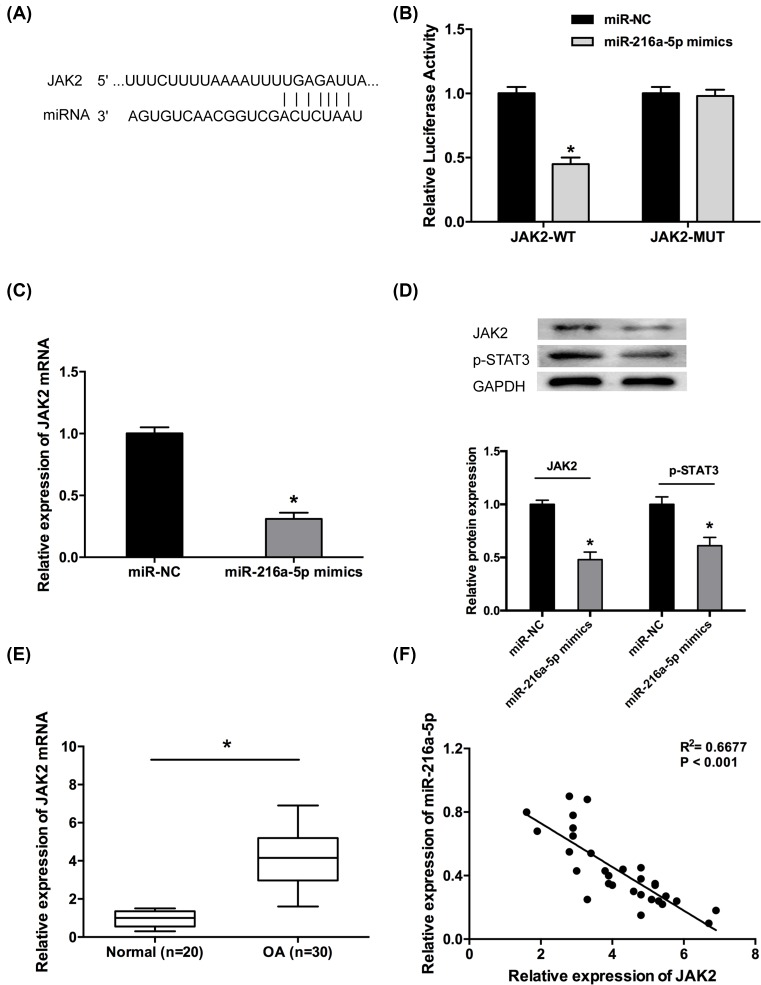
JAK2 was a direct target of miR-216a-5p in OA chondrocytes (**A**) A putative miR-216a-5p target site in the 3′-UTR of JAK2 mRNA was predicted using bioinformatics analysis. (**B**) Luciferase reporter assay demonstrated that miR-216a-5p mimics significantly decreased the luciferase activity of JAK2-WT in OA chondrocytes. (**C**) MiR-216a-5p mimics decreased the relative expression of JAK2 mRNA in OA chondrocytes. (**D**) The effect of miR-216a-5p mimics on the protein expression of JAK2, p-STAT3 in OA chondrocytes. (**E**) The relative expression of JAK2 mRNA was significantly up-regulated in OA patients compared with normal patients. (**F**) The inverse relationship was observed between miR-216a-5p and JAK2 expression in OA cartilage tissues. Data are shown as mean ± S.D. ^*^P<0.05.

MiRNAs influence biological functions by inversely regulating target genes. Through online bioinformatics software, we found JAK2 was a potential downstream target of miR-216a-5p (Figure 5A). Luciferase reporter assay showed that miR-216a-5p mimics significantly decreased the luciferase activity of JAK2-WT reporter vector but not the JAK2-MUT reporter vector (Figure 5B, *P*<0.05). In addition, miR-216a-5p mimics remarkably inhibited the mRNA and protein expression of JAK2 in OA chondrocytes (Figure 5C,D, *P*<0.05). Previous studies showed that JAK2/STAT3 signaling pathway play important roles in osteoarthritis. In the present study, miR-216a-5p mimics also led to significantly decrease of p-STAT3, which decreasing the activation of JAK2/STAT3 signaling pathway (Figure 5D, *P*<0.05). Moreover, qRT-PCR assay revealed that the expression of JAK2 was up-regulated in OA patients, and negatively correlated with the expression of miR-216a-5p in OA samples (Figure 5E,F, *P*<0.05).

### DANCR regulated the JAK2/STAT3 expression through miR-216a-5p in OA chondrocytes

**Figure 6:**
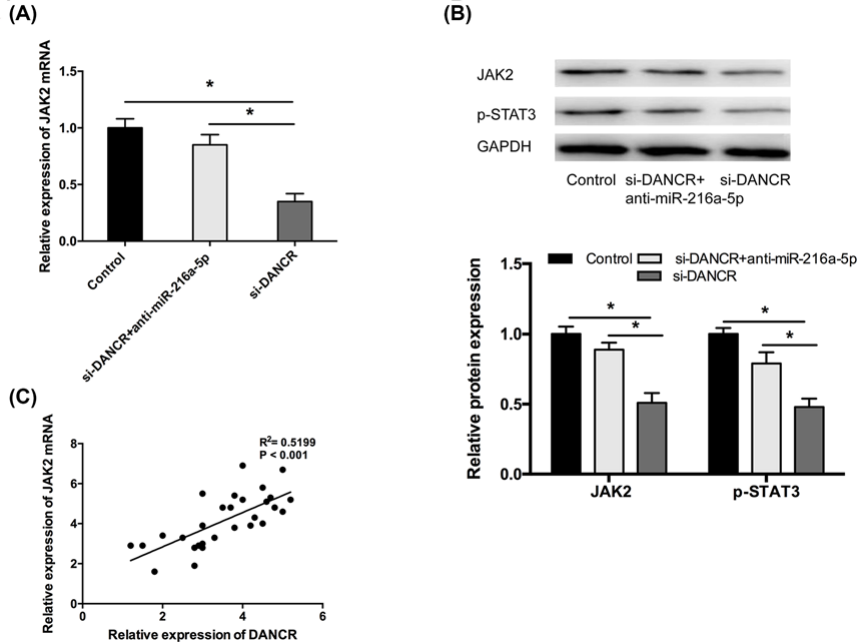
DANCR regulated the JAK2/STAT3 pathway through miR-216a-5p in OA chondrocytes (**A**) DANCR inhibition markedly reduced the expression of JAK2 mRNA in OA chondrocytes, which was obviously reversed by the introduction of miR-216a-5p inhibitors. (**B**) The influence of DANCR inhibition on the protein expression of JAK2 and p-STAT3 in OA chondrocytes was abolished by miR-216a-5p inhibitors. (**C**) The positive relationship was observed between DANCR and JAK2 expression in OA cartilage tissues. Data are shown as mean ± SD. ^*^P<0.05.

Based on the above, we further investigated whether DANCR involved in the miR-216a-5p/JAK2/STAT3 signaling pathway in OA chondrocytes. The results showed that DANCR inhibition markedly decreased the mRNA and protein expression of JAK2 in OA chondrocytes, which was obviously reversed by the introduction of miR-216a-5p inhibitors (Figure 6A,B, *P*<0.05). Furthermore, pearson’s correlation analysis revealed that DANCR expression was positively correlated with the JAK2 expression in OA patients (Figure 6C, *P*<0.05). Moreover, DANCR inhibition led to significant decrease in the protein expression of p-STAT3, while miR-216a-5p inhibitors could abolish the inhibitory effects (Figure 6B, *P*<0.05). Taken together, these results suggested that DANCR regulated the JAK2/STAT3 expression through miR-216a-5p in OA chondrocytes.

## Discussion

OA is a degenerative disease of articular cartilage and the main pathological feature is cartilage destruction. Since the underlying mechanism of OA is not fully learned, there is no fundamental therapy [15]. Recently, more and more studies have demonstrated that lncRNAs play important roles in the development and progression of OA [16]. Several lncRNAs were identified to participate in OA progression, including HOTAIR [17], FAS-AS1 [18], SNHG5 [19] and MEG3 [20]. LncRNA DANCR was first identified in hepatocellular carcinoma, and functioned as an oncogene in multiple cancers [21]. Moreover, DANCR could promote cell proliferation and chondrogenic differentiation of human synovium-derived mesenchymal stem cells [22]. In the present study, we found that DANCR was significantly increased in OA patients. DANCR inhibition decreased the proliferation and increased apoptosis of OA chondrocytes. Cartilage degradation can be promoted via proliferation and inflammatory factors of chondrocytes [23]. It was reported that increased concentrations of inflammatory factors, including IL-6 and IL-8, promote OA progression by enhancing cartilage degradation [24]. In our study, we also found that inhibition of DANCR significantly inhibited the expression levels of IL-6 and IL-8 in OA chondrocytes. These results revealed that lncRNA DANCR promoted OA progression through increasing inflammation, cell proliferation and decreasing cell apoptosis.

However, the underlying mechanism of the involvement of DANCR in OA remains unclear. An increasing number of studies demonstrated that lncRNAs functioned as a ceRNA of miRNAs, and in turn regulating their biological functions in a variety of diseases. LncRNA DANCR was also found to act as a ceRNA of several miRNAs. For instance, Jiang et al. reported that DANCR promoted tumor progression and cancer stemness features in osteosarcoma via competitively binding to miR-33a-5p [25]. Emerging evidence supported that lncRNAs might possess the novel regulatory mechanism to act as sponges for miRNAs during OA pathogenesis. For example, Wang et al. indicated that lncRNA UCA1 promoted chondrocytes cell proliferation and collagen expression through acting as a molecular sponge of miR-204-5p [26]. Our study identified miR-216a-5p as a potential target of DANCR. Further luciferase activity reporter assay and RIP assay validated that DANCR directly interacted with miR-216a-5p in OA chondrocytes. DANCR regulated inflammation, proliferation and apoptosis of OA chondrocytes through sponging miR-216a-5p.

A series of previous studies showed that miRNAs exerted their functional roles by regulating the expression of endogenous targets [27]. For example, Liu et al. reported that miR-216a-5p functioned as a tumor suppressor in uveal melanoma through the inhibition of HK2 [28]. In the present study, it is verified that JAK2 was a target gene of miR-216a-5p by luciferase activity reporter assay, and miR-216a-5p mimics markedly repressed JAK2 expression in OA chondrocytes.

JAK2 is an important tumor oncogene for several cancers including gastric cancer and lung cancer [29,30]. Recently, lots study showed that JAK2/STAT3 pathway play important roles in osteoarthritis. For example, Yao et al. showed that DUSP19 regulated IL-1β-induced apoptosis and MMPs expression in rat chondrocytes through JAK2/STAT3 signaling pathway [31]. Xu et al. found that Danshen attenuated cartilage injuries in osteoarthritis *in vivo* and *in vitro* by activating JAK2/STAT3 and AKT pathways [32]. In the present study, JAK2 expression was up-regulated in OA patients. MiR-216a-5p mimics decreased the protein expression of p-STAT3, which decreasing the activation of JAK2/STAT3 signaling pathway in OA chondrocytes. It was also found that DANCR was involved in miR-216a-5p/JAK2/STAT3 axis in chondrocytes. Thus, we indicated that DANCR exerted its pro-inflammation, pro-proliferation, and anti-apoptosis roles by regulating miR-216a-5p/JAK2/STAT3 signaling pathway in OA chondrocytes.

In conclusion, lncRNA DANCR was up-regulated in OA cartilage tissues. DANCR suppression inhibited inflammation, cell proliferation, and promoted apoptosis in OA chondrocytes through miR-216a-5p/JAK2/STAT3 signaling pathway, indicating that DANCR might be a useful biomarker and potential therapeutic target for OA treatment.

## Abbreviations

DANCR: differentiation antagonizing non-protein coding RNA
DANCR-MUT: mutated DANCR 3′-UTR
DANCR-WT: wild-type DANCR 3′UTR
IL: interleukin
LncRNA: long non-coding RNA
OA: osteoarthritis
qRT-PCR: quantitative real-time PCR
RIP: RNA immunoprecipitation

## References

[1] Hussain S.M., Neilly D.W., Baliga S., Patil S. and Meek R. (2016) Knee osteoarthritis: a review of management options. Scott. Med. J. 61, 7–16 10.1177/0036933015619588 27330013

[2] Pereira D., Severo M., Santos R.A., Barros H., Branco J., Lucas R. (2016) Knee and hip radiographic osteoarthritis features: differences on pain, function and quality of life. Clin. Rheumatol. 35, 1555–1564 10.1007/s10067-015-3087-7 26445941

[3] Taylor N. (2017) Nonsurgical management of osteoarthritis knee pain in the older adult. Clin. Geriatr. Med. 33, 41–51 10.1016/j.cger.2016.08.004 27886697

[4] Ponting C.P., Oliver P.L. and Reik W. (2009) Evolution and functions of long noncoding RNAs. Cell 136, 629–641 10.1016/j.cell.2009.02.006 19239885

[5] Xu J. and Cao X. (2018) Long noncoding RNAs in the metabolic control of inflammation and immune disorders. Cell Mol. Immunol. 10.1038/s41423-018-0042-y 29795339 PMC6318285

[6] Fatica A. and Bozzoni I. (2014) Long non-coding RNAs: new players in cell differentiation and development. Nat. Rev. Genet. 15, 7–21 10.1038/nrg3606 24296535

[7] Dey B.K., Mueller A.C. and Dutta A. (2014) Long non-coding RNAs as emerging regulators of differentiation, development, and disease. Transcription 5, e944014 10.4161/21541272.2014.944014 25483404 PMC4581346

[8] Archer K., Broskova Z., Bayoumi A.S., Teoh J.P., Davila A., Tang Y. (2015) Long non-coding RNAs as master regulators in cardiovascular diseases. Int. J. Mol. Sci. 16, 23651–23667 10.3390/ijms161023651 26445043 PMC4632719

[9] Liu X., Hao Y., Yu W., Yang X., Luo X., Zhao J. (2018) Long non-coding RNA emergence during renal cell carcinoma tumorigenesis. Cell. Physiol. Biochem. 47, 735–746 10.1159/000490026 29794462

[10] Fu M., Huang G., Zhang Z., Liu J., Zhang Z., Huang Z. (2015) Expression profile of long noncoding RNAs in cartilage from knee osteoarthritis patients. Osteoarthritis Cartilage 23, 423–432 10.1016/j.joca.2014.12.001 25524778

[11] Li Y.F., Li S.H., Liu Y. and Luo Y.T. (2017) Long noncoding RNA CIR promotes chondrocyte extracellular matrix degradation in osteoarthritis by acting as a sponge For Mir-27b. Cell. Physiol. Biochem. 43, 602–610 10.1159/000480532 28934732

[12] Yuan S.X., Wang J., Yang F., Tao Q.F., Zhang J., Wang L.L. (2016) Long noncoding RNA DANCR increases stemness features of hepatocellular carcinoma by derepression of CTNNB1. Hepatology 63, 499–511 10.1002/hep.27893 25964079

[13] Zhang L., Yang C., Chen S., Wang G., Shi B., Tao X. (2017) Long noncoding RNA DANCR is a positive regulator of proliferation and chondrogenic differentiation in human synovium-derived stem cells. DNA Cell Biol. 36, 136–142 10.1089/dna.2016.3544 27982693

[14] Dahlin R.L., Meretoja V.V., Ni M., Kasper F.K. and Mikos A.G. (2014) Chondrogenic phenotype of articular chondrocytes in monoculture and co-culture with mesenchymal stem cells in flow perfusion. Tissue Eng. Part A 20, 2883–2891 10.1089/ten.tea.2014.0107 24745375 PMC4229696

[15] Goldring M.B. and Berenbaum F. (2015) Emerging targets in osteoarthritis therapy. Curr. Opin. Pharmacol. 22, 51–63 10.1016/j.coph.2015.03.004 25863583 PMC4470796

[16] Pearson M.J. and Jones S.W. (2016) Review: long noncoding RNAs in the regulation of inflammatory pathways in rheumatoid arthritis and osteoarthritis. Arthritis Rheumatol 68, 2575–2583 10.1002/art.39759 27214788 PMC5347907

[17] Dou P., Hu R., Zhu W., Tang Q., Li D., Li H. (2017) Long non-coding RNA HOTAIR promotes expression of ADAMTS-5 in human osteoarthritic articular chondrocytes. Pharmazie 72, 113–117 29441864 10.1691/ph.2017.6649

[18] Zhu J.K., He T.D., Wei Z.X. and Wang Y.M. (2018) LncRNA FAS-AS1 promotes the degradation of extracellular matrix of cartilage in osteoarthritis. Eur. Rev. Med. Pharmacol. Sci. 22, 2966–2972 29863238 10.26355/eurrev_201805_15051

[19] Shen H., Wang Y., Shi W., Sun G., Hong L. and Zhang Y. (2018) LncRNA SNHG5/miR-26a/SOX2 signal axis enhances proliferation of chondrocyte in osteoarthritis. Acta Biochim. Biophys. Sin. (Shanghai) 50, 191–198 10.1093/abbs/gmx141 29409014

[20] Su W., Xie W., Shang Q. and Su B. (2015) The long noncoding RNA MEG3 is downregulated and inversely associated with VEGF levels in osteoarthritis. Biomed. Res. Int. 2015, 356893 10.1155/2015/356893 26090403 PMC4454735

[21] Thin K.Z., Liu X., Feng X., Raveendran S. and Tu J.C. (2018) LncRNA-DANCR: a valuable cancer related long non-coding RNA for human cancers. Pathol. Res. Pract. 214, 801–805 10.1016/j.prp.2018.04.003 29728310

[22] Zhang L., Sun X., Chen S., Yang C., Shi B., Zhou L. (2017) Long noncoding RNA DANCR regulates miR-1305-Smad 4 axis to promote chondrogenic differentiation of human synovium-derived mesenchymal stem cells. Biosci. Rep. 37, pii: BSR20170347 10.1042/BSR20170347 PMC5520215 28674107

[23] Goldring M.B. (2012) Chondrogenesis, chondrocyte differentiation, and articular cartilage metabolism in health and osteoarthritis. Ther. Adv. Musculoskelet Dis. 4, 269–285 10.1177/1759720X12448454 22859926 PMC3403254

[24] Xu L., Peng Q., Xuan W., Feng X., Kong X., Zhang M. (2016) Interleukin-29 enhances synovial inflammation and cartilage degradation in osteoarthritis. Mediators Inflamm. 2016, 9631510 10.1155/2016/9631510 27433031 PMC4940582

[25] Jiang N., Wang X., Xie X., Liao Y., Liu N., Liu J. (2017) lncRNA DANCR promotes tumor progression and cancer stemness features in osteosarcoma by upregulating AXL via miR-33a-5p inhibition. Cancer Lett. 405, 46–55 10.1016/j.canlet.2017.06.009 28642170

[26] Wang G., Bu X., Zhang Y., Zhao X., Kong Y., Ma L. (2017) LncRNA-UCA1 enhances MMP-13 expression by inhibiting miR-204-5p in human chondrocytes. Oncotarget 8, 91281–91290 29207643 10.18632/oncotarget.20108 PMC5710923

[27] Macfarlane L.A. and Murphy P.R. (2010) MicroRNA: biogenesis, function and role in cancer. Curr. Genomics 11, 537–561 10.2174/138920210793175895 21532838 PMC3048316

[28] Liu Y., Huo Y., Wang D., Tai Y., Li J., Pang D. (2018) MiR-216a-5p/Hexokinase 2 axis regulates uveal melanoma growth through modulation of Warburg effect. Biochem. Biophys. Res. Commun. 501, 885–892 10.1016/j.bbrc.2018.05.069 29763606

[29] Zhao J.M., Cheng W., He X.G. (2018) Long non-coding RNA PICART1 suppresses proliferation and promotes apoptosis in lung cancer cells by inhibiting JAK2/STAT3 signaling. Neoplasma 10.4149/neo_2018_171130N778 29940776

[30] Tao Y., Yang S., Wu Y. (2017) MicroRNA-216a inhibits the metastasis of gastric cancer cells by targeting JAK2/STAT3-mediated EMT process. Oncotarget 8, 88870 10.18632/oncotarget.21488 29179483 PMC5687653

[31] Yao Z.Z., Hu A.X. and Liu X.S. (2017) DUSP19 regulates IL-1β-induced apoptosis and MMPs expression in rat chondrocytes through JAK2/STAT3 signaling pathway. Biomed. Pharmacother. 96, 1209–1215 10.1016/j.biopha.2017.11.097 29174854

[32] Xu X., Lv H., Li X. (2018) Danshen attenuates cartilage injuries in osteoarthritis *in vivo* and *in vitro* by activating JAK2/STAT3 and AKT pathways. Exp. Anim. 67, 127–137 10.1538/expanim.17-0062 29093428 PMC5955744

